# Preparation and Evaluation of Tadalafil-Loaded Nanoemulgel for Transdermal Delivery in Cold-Induced Vasoconstriction: A Potential Therapy for Raynaud’s Phenomenon

**DOI:** 10.3390/pharmaceutics17050596

**Published:** 2025-05-01

**Authors:** Shery Jacob, Jamila Ojochenemi Abdullahi, Shahnaz Usman, Sai H. S Boddu, Sohaib Naseem Khan, Mohamed A. Saad, Anroop B Nair

**Affiliations:** 1 Department of Pharmaceutical Sciences, College of Pharmacy, Gulf Medical University, Ajman 4184, United Arab Emirates; 2022mdd02@mygmu.ac.ae (J.O.A.); dr.abdullatif@gmu.ac.ae (M.A.S.); 2 Department of Pharmaceutics, RAK College of Pharmacy, RAK Medical and Health Sciences University, Ras Al Khaimah 11172, United Arab Emirates; shahnazgauhar@gmail.com; 3 Department of Pharmaceutics, Faculty of Pharmacy, Salim Habib University, Karachi 74900, Pakistan; 4 Department of Pharmaceutical Sciences, College of Pharmacy and Health Sciences, Ajman University, Ajman 346, United Arab Emirates; s.boddu@ajman.ac.ae (S.H.S.B.); sohaib.khan@ajman.ac.ae (S.N.K.); 5 Center of Medical and Bio-Allied Health Sciences Research, Ajman University, Ajman 346, United Arab Emirates; 6 Department of Pharmaceutical Sciences, College of Clinical Pharmacy, King Faisal University, Al-Ahsa 31982, Saudi Arabia; anair@kfu.edu.sa

**Keywords:** Raynaud’s phenomenon, tadalafil, nanoemulsion, nanoemulgel, pharmacodynamics, pharmacokinetics

## Abstract

**Background/Objectives:** Raynaud’s phenomenon (RP) is characterized by an exaggerated vasoconstrictive response of small blood vessels in the fingers and toes to cold or stress. Oral therapy with tadalafil (TDL), a phosphodiesterase-5 inhibitor, is limited by systemic side effects and reduced patient compliance. This study aimed to develop and evaluate a TDL-loaded nanoemulgel for transdermal delivery as a non-invasive treatment alternative for cold-induced vasoconstriction. **Methods:** TDL-loaded nanoemulsions were prepared using the aqueous titration method with cinnamon oil as the oil phase and Cremophor RH40 and Transcutol as the surfactant–cosurfactant system. The optimized nanoemulsion was incorporated into a carbopol-based gel to form a nanoemulgel. The formulation was characterized for droplet size, morphology, thermodynamic stability, rheological properties, in vitro drug release, skin permeation, and pharmacokinetic behavior. Infrared thermography was employed to assess in vivo efficacy in cold-induced vasoconstriction models. **Results:** The optimized TDL nanoemulsion exhibited a spherical morphology, a nanoscale droplet size, and an enhanced transdermal flux. The resulting nanoemulgel displayed suitable physicochemical and rheological properties for topical application, a short lag time (0.7 h), and a high permeability coefficient (Kp = 3.59 × 10^−2^ cm/h). Thermal imaging showed significant vasodilation comparable to standard 0.2% nitroglycerin ointment. Pharmacokinetic studies indicated improved transdermal absorption with a higher C_max_ (2.13 µg/mL), a prolonged half-life (t_1/2_ = 16.12 h), and an increased AUC_0–24_ compared to an oral nanosuspension (*p* < 0.001). **Conclusions:** The developed TDL nanoemulgel demonstrated effective transdermal delivery and significant potential as a patient-friendly therapeutic approach for Raynaud’s phenomenon, offering an alternative to conventional oral therapy.

## 1. Introduction

Raynaud’s phenomenon (RP) is a chronic vasospastic condition affecting the extremities and is characterized by a triad of pallor, bluish discoloration (cyanosis), and redness. It occurs due to blood vessel constriction in response to cold exposure or emotional stress and can sometimes extend to the lips, earlobes, nipples, and nose tip [1]. RP triggers a distinctive sequence of color changes in the digits—pallor resulting from vasoconstriction, bluish discoloration due to diminished oxygenated blood flow, and erythema upon reperfusion and hyperemia. The prolonged state of diminished oxygen supply leads to gangrene, ulcers, and ultimately death of tissues [1,2]. RP can be classified as either primary/idiopathic, with no known cause, or secondary, linked to conditions such as autoimmune disorders such as systemic sclerosis, vascular diseases, and neurological disorders. The reported prevalence of RP ranges from 2.1% to 22.4%, varying across studies due to factors such as geographic location, ethnicity, and differences in diagnostic criteria [3]. The precise origin of RP is unknown, but genetic and hormonal factors—particularly estrogen—may play a role and may explain the increased prevalence of RP in females compared to males [1]. Its pathogenesis arises from a complex interaction among the vascular wall, nerves, hormones, and humoral factors, resulting in an imbalance between vasoconstriction and vasodilation [4]. There is no known cure for RP, but management involves pharmacological and nonpharmacological modalities. Nonpharmacological management is recommended for all patients and mainly involves avoiding triggers, i.e., cold exposure and stressors. Pharmacological management includes the use of calcium channel blockers (first-line therapy). The dihydropyridine class of calcium antagonists (e.g., nifedipine, amlodipine) is preferred for use in RP, with nifedipine as the most employed as the drug of choice [5]. Angiotensin-converting enzyme inhibitors, angiotensin receptor antagonists, and selective serotonin reuptake inhibitors are additionally utilized in the treatment of uncomplicated RP [1]. In cases of complicated RP, oral PDE5 (phosphodiesterase-5) inhibitors and IV prostanoids are employed. Endothelin-1 receptor antagonists such as bosentan, botulinum toxin (Botox), and surgical therapy like sympathectomy are other management options used in severe and refractory cases of RP [4].

The role of PDE5 inhibitors in RP has been increasingly evaluated, with results from a meta-analysis showing a moderate but significant improvement in RP symptoms [6]. Tadalafil (TDL), a selective PDE-5 inhibitor, increases cGMP levels, leading to vasodilation and smooth muscle relaxation. It is used to treat erectile dysfunction [7], pulmonary arterial hypertension [8], and benign prostatic hyperplasia [9]. A clinical trial for the use of TDL in RP was undertaken with promising results albeit a small sample size [10]. TDL has also been evaluated as an add-on therapy in secondary resistant RP and was observed to improve the symptoms of RP, preventing and healing digital ulcers and enhancing overall quality of life [11].

TDL belongs to BCS class II drugs with low water solubility (3.2 µg/mL), leading to variable absorption and an undetermined absolute bioavailability [12]. TDL has a longer half-life (15–17 h), higher selectivity for PDE-5, and lower affinity for PDE-6, which reduces the risk of visual side effects commonly associated with other PDE-5 inhibitors [13]. It also has the slowest absorption rate among PDE-5 inhibitors, which limits the effectiveness of its oral formulation [13]. Its optimum log *p* value of 2.89, relatively low molecular weight (389.4 g/mol), and low dose make TDL a potential candidate for alternative drug delivery methods, such as transdermal administration [14].

Transdermal and dermal delivery systems are thought to be an appealing alternative route for drug administration, offering both local and/or systemic activity, in comparison to oral and parenteral delivery systems [15]. The transdermal delivery system (TDDS) is advantageous as it bypasses hepatic first-pass metabolism, providing a constant plasma drug concentration, increasing bioavailability, reducing dosing frequency, and decreasing systemic side effects [16]. Moreover, it promotes tolerability and compliance by being non-invasive, painless, easy to administer, minimizing stomach irritation, overcoming unappealing taste, and providing a good option for individuals with dysphagia [17]. The non-viable uppermost skin layer, known as the stratum corneum, acts as a physiological barrier, which generally restricts drug absorption and permeability in topical and transdermal delivery systems [18]. As the skin’s natural barrier properties pose challenges for TDDS, efforts to enhance transdermal delivery are ongoing, with current methods focused on increasing penetration into the skin layers through the use of penetration enhancers, such as chemical agents, techniques like iontophoresis, sonophoresis, electroporation, microneedles, thermophoresis, or thermal ablation, and drug delivery carriers like nanoparticles, albeit with certain drawbacks [19]. These limitations include constraints in drug applicability, potential for skin irritation, limited skin penetration depth, risk of infection, safety concerns, and the need for specialized equipment.

Transdermal delivery of TDL has been explored in various studies. In one attempt, TDL-loaded nanostructured lipid carriers have been fabricated to enhance its skin permeability upon the inclusion of ethyl alcohol and limonene [20]. However, the inclusion of ethyl alcohol in transdermal formulation has the potential to induce skin dryness, irritation, or contact dermatitis, both of which can aggravate the condition of RP [21]. In another attempt, formulation using Carbopol 940 gel with hydroxypropyl-β-cyclodextrin (HPβCD) and oleic acid confirmed enhanced TDL solubility and permeability [13]. Though the inclusion of HPβCD in transdermal formulations improves solubility and reduces irritation, its inherent hydrophilicity limits its permeability, often necessitating additional enhancers [22]. It also presents challenges such as drug retention and higher cost. Recently, a transdermal formulation of TDL was developed and evaluated using Strat-M^®^ membrane [23]. The formulation, which utilized hexadecyltrimethylammonium bromide (HDTMA-Br) with dipropylene glycol, showed good skin permeability and remained stable over 12 months. However, HDTMA-Br is not currently approved for transdermal pharmaceutical products by major regulatory agencies due to safety and irritation concerns in human use. On the other hand, nanoemulsion-based oral jellies loaded with TDL demonstrated improved bioavailability compared to the free form of TDL [24]. However, the observed maximum plasma concentration (C_max_) and area under the curve (AUC) were relatively low, suggesting relatively poor absorption through the oral route. A recent study optimized a TDL nano-ointment with a particle size of 208 nm, polydispersity index (PDI) of 0.404, and zeta potential of 31.0 mV, indicating good stability and uniform dispersion [25]. In vitro and ex vivo studies demonstrated that the nano-ointment provided sustained, controlled release of TDL following zero-order kinetics, with better drug permeation correlation than the TDL cream. Although the formulation was proposed for RP management, no pharmacodynamic or pharmacokinetic studies were conducted to substantiate its in vivo therapeutic efficacy. Based on all these observations, the development of a new transdermal formulation of TDL is warranted, with a focus on ensuring safety and non-irritancy for use in patients with RP.

Nanovesicles demonstrate significant promise in TDDS by virtue of their unique properties like small size, high surface area to volume ratio, potential to encapsulate both hydrophilic and lipophilic drugs, skin permeation without significantly changing the skin’s physiological and functional characteristics [26]. Nanovesicles are often favored over nanoparticles in TDDS due to their superior skin absorption, compatibility with biological systems, adaptability, increased stability, extended drug release, and ability to target specific areas. Drug encapsulation in lipid nanocarriers presents a viable strategy for effective delivery, with predictable characteristics that improve bioavailability and reduce undesirable side effects [27]. Among various lipid-based nanosystems, nanoemulsions are favored for transdermal drug delivery because of their unique characteristics and advantages over other lipid nanovesicles [28]. Nanoemulsions can enhance skin penetration of drugs by reducing the barrier function of the skin, improving drug partitioning into the skin, and enhancing drug retention in the skin. Thus, nanoemulsions can be considered an ideal platform for transdermal therapy due to their advantages, such as prolonged drug release, enhanced stability, versatility, biocompatibility, and ease of scale-up [29].

Nanoemulgel is an advanced drug delivery system that combines nanoemulsion droplets within an aqueous gel matrix, creating a stable, non-greasy, and thixotropic formulation. This system enhances drug penetration, permeability, and absorption, providing prolonged drug release, and improved stability making it ideal for transdermal drug delivery [30]. Furthermore, gel formulations are convenient to apply, rarely cause skin irritation, and thus enhance patient compliance [31].

Even though TDL has been recommended as an oral add-on therapy for RP, research on its nanoformulation for transdermal delivery remains limited, highlighting the need for further studies. Therefore, the current research aimed to develop and evaluate a TDL-loaded nanoemulgel for transdermal delivery to enhance bioavailability and assess its therapeutic efficacy in managing RP using a cold-induced vasoconstriction rat model. The selected TDL-loaded nanoemulgel was evaluated for TDL release, transdermal permeation, and in vivo efficacy in the rat model.

## 2. Materials and Methods

### 2.1. Materials

TDL was donated by Julphar Pharmaceuticals, Ras Al Khaimah, UAE. Cremophor RH 40 was purchased from Sigma Aldrich (St. Louis, MO, USA), and Carbopol 934 was procured from SRL CHEM (Mumbai, India). Diethylene glycol monoethyl ether (Transcutol/Carbitol) 98% was obtained from Loba Chemie (Mumbai, India). Cinnamon oil was procured from AVD Organics (Mumbai, India). All other chemicals, including ketamine and xylazine used for the animal studies, were employed in their received form and were of reagent grade or higher quality.

### 2.2. HPLC Determination of Tadalafil

Quantification of TDL was carried out in an HPLC system (Shimadzu, Kyoto, Japan) with an LC-20AD pump, DGU-20A 3 degasser, SPD-20A UV-VIS detector, and a Restex Force C18 column (5 μm, 150 × 4.6 mm). The mobile phase consisted of acetonitrile and water in a 45%:55% (*v*/*v*) ratio with 0.1% trifluoracetic acid. The flow rate of the mobile phase was fixed at 1 mL/min, and the temperature of the column was maintained at 40 °C. After injecting 20 µL samples, the chromatogram was recorded (wavelength of 285 nm). For TDL concentration analysis, plasma was subjected to HPLC after protein precipitation with 100 µL of 1N HCl and 2 mL of diethyl ether, followed by vortexing for 10 min. Subsequently, the samples were centrifuged at 4000 rpm for 15 min, after which the supernatant was collected, filtered, and dried. The residue obtained was then mixed with the mobile phase, filtered (0.45 µm syringe filter), and further injected into the HPLC column. The drug level of TDL between 0.0625–12 µg/mL showed good linearity (R^2^ = 0.9989).

### 2.3. Screening of Oil

The equilibrium solubility of TDL in selected oils was measured [32] by adding an excess quantity of the drug to 1 mL of oils (Miglyol^®^ 812 N, Miglyol^®^ 840, Miglyol^®^ 829, triacetin, isopropyl myristate, castor oil, oleic acid, cinnamon oil, N-methyl pyrrolidone) separately in 1.5 mL stoppered glass vials and mixed by a vortex mixer. Samples were further placed at room temperature (25 ± 1 °C) on a shaker (Incu-Shaker, Benchmark Scientific, Edison, NJ, USA) at 180 rpm for 72 h. The mixture was further centrifuged for 15 min at 3000 rpm. The upper supernatant was removed, and the amount of drug was determined using UV analyzer (Jenway, Staffs, UK) at a lambda max of 284 nm. The data presented in Table 1 are the mean values and standard deviations (SDs) for each oil sample, based on three replicates.

### 2.4. Screening of Surfactants and Cosurfactants

The solubility screening of TDL in various surfactants (Tween 80, Span 80, Cremophor RH 40, PEG-400, propylene glycol, isopropyl alcohol, and Transcutol) was performed following the same procedure as the oil screening. The results presented in the article are the mean values and standard deviations (SDs) for surfactant and cosurfactant samples, based on three replicates.

### 2.5. Construction of Pseudoternary Phase Diagram

To determine the nanoemulsion area, the pseudoternary phase diagram was generated by systematically altering the surfactant, cosurfactant, oil, and aqueous phase ratios. The surfactant and cosurfactant were blended in various weight ratios (3:1, 2:1, 1:1, 1:0, 1:0.25, 1:0.5, and 1:0.7) to create different Smix combinations. Oil was then incorporated into the Smix system and the aqueous titration technique was utilized to create the phase diagram utilizing a vortex mixer (Model: REMI CM-101, Mumbai, India) at 25 ± 1 °C. To ensure comprehensive coverage of possible formulations, the oil-to-water (o/w) ratios were varied across a wide range (9:1, 8:2, 7:3, 6:4, 5:5). The resulting mixtures were visually assessed to identify the nanoemulsion region, characterized by clear, transparent, and easily flowable formulations. Mixtures that appeared turbid or showed phase separation were considered outside the nanoemulsion region. The final pseudoternary phase diagram was plotted with three axes representing the Smix system, aqueous phase, and oil phase. This diagram provided a visual representation of the regions where stable nanoemulsions formed, offering critical insights into the optimal formulation parameters for transdermal delivery systems.

### 2.6. Preparation of Tadalafil-Loaded Nanoemulsion

**Figure 1:**
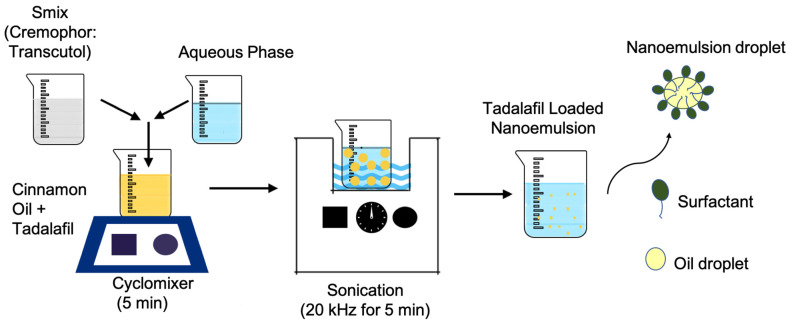
Schematic representation of tadalafil-loaded nanoemulsion preparation.

The schematic representation of nanoemulsion preparation is shown in Figure 1. TDL was first dissolved in the selected oil phase (cinnamon oil) by gentle mixing. Deionized water was then added as the aqueous phase, followed by the incorporation of Smix (Cremophor 40 and Transcutol) in a 1:0.7 *v*/*v* ratio. The composition was blended using a cyclomixer (REMI CM-101, Mumbai, India) for 5 min to ensure uniformity. Later, the mixture was then subjected to ultrasonication in a bath sonicator (Model: Nickel-Electro, SW3H, Basel, Switzerland) at room temperature (25 ± 1 °C) for 5 min at a frequency of 20 kHz to form nanodroplets and achieve a stable nanoemulsion. All experiments were performed in triplicate, and the resulting data are presented as the mean values with standard deviations (SDs).

### 2.7. Characterization of TDL-Loaded Nanoemulsion

#### 2.7.1. Drug Content

A precisely measured 1 mL of TDL-loaded nanoemulsion was placed in a polypropylene centrifuge tube, followed by the gradual addition of the mobile phase. The mixture was agitated for 1 h using a mechanical shaker (Incu-Shaker, Benchmark Scientific, Edison, NJ, USA) to achieve a uniform solution. It was then centrifuged (MiniSpin, Eppendorf, Germany) at 4280× *g* for 10 min. The resultant supernatant (1 mL) was filtered with syringe filter (pore size, 0.22 µm). The drug content was determined by HPLC after appropriate dilution. All results are expressed as the mean ± standard deviation (n = 3) for each sample.

#### 2.7.2. Percentage Transmittance and pH

The transmittance of nanoemulsions was measured using a UV spectrophotometer (Jenway, Staffs, UK). The instrument was calibrated by setting the transmittance to 100% with a transparent cuvette containing double-distilled water as a blank at 400 nm. Nanoemulsion samples were then placed in the cuvette, and their percentage transmittance was recorded. The formulations (S1–S7) were tested for pH at 25 ± 1 °C with the help of pH meter (Jen-way 3510, Staffs, UK). All the experiments were conducted in triplicate and the data generated were presented as the mean values and SDs.

#### 2.7.3. Dilution Potential

The dilution test was conducted to examine the potential phase inversion of the optimized nanoemulsions. To assess the dilution potential, nanoemulsion (1 mL) was diluted tenfold with deionized water in a test tube, and no occurrence of phase separation was observed. Data are reported as the mean ± SD. from three independent experiments.

#### 2.7.4. Droplet Size and Zeta Potential

The average particle size along with the PDI, as well as zeta potential of nanoemulsion was evaluated by Horiba Zetasizer (model, SZ-100, Kyoto, Japan). Approximately 2 mL of test samples were placed in the chamber and analysed for particle size and zeta potential using Horiba SZ-100 software (Z-type, version 2.20, Kyoto, Japan). All experiments were carried out in triplicate, and the resulting data are presented as the mean values with standard deviations (SD).

#### 2.7.5. Viscosity

The viscosity of the selected nanoemulsions (S1–S7) was measured at various angular velocities at 25 °C using an Atago viscometer (Visco-895, Tokyo, Japan) [33]. Data are expressed as the mean ± SD from triplicate experiments.

#### 2.7.6. Thermodynamic Stability Studies

The purpose of this study was to evaluate the physical stability of selected formulations through various tests. In the heating–cooling cycle, the nanoemulsions underwent six cycles between 4 °C and 45 °C, with a storage period of at least 48 h at each temperature, to evaluate their stability under extreme temperature fluctuations, using refrigerator (LG Electronics, New Delhi, India) and Hot air oven (SCT-Convect-1, Sci-Chem, Mumbai, India). The centrifugation test involved spinning the formulations at 3500 rpm for 30 min to assess for any phase separation, performed with a high-speed centrifuge (MiniSpin, Eppendorf, Hamburg, Germany). Additionally, a freeze–thaw cycle test was performed with three cycles between −21 °C and 25 °C, where the formulations were placed at a particular temperature for at least 48 h to observe any signs of instability, using a deep freezer (Forma, Thermo Fisher Scientific, Waltham, MA, USA) and at room temperature. All the experiments were conducted in triplicate and the data generated were presented as the mean values and SDs.

#### 2.7.7. Fourier-Transform Infrared (FTIR) Spectroscopy

The potential drug–formulation component interactions and the stability of TDL in formulation ingredients were assessed using FTIR spectroscopy. IR spectra of TDL, a blank nanoemulsion, and a drug-loaded nanoemulsion were collected for samples stored for 30 days at 25 ± 0.2 °C and 75% ± 5% RH [19]. After the storage period, the samples were analyzed using a Shimadzu FTIR spectrometer (IRXross, Shimadzu, Kyoto, Japan) equipped with a diamond attenuated total reflectance accessory. This facilitates direct measurement by pressing the sample against a diamond prism and accommodates various sample types. Scanning was performed in the range of 145 to 4000 cm^−1^, with ~25 scans/sample and 2 cm^−1^ resolution. The spectra of TDL, the blank nanoemulsion, and the drug-loaded nanoemulsion samples were compared to identify any variations in the characteristic peaks of the drug, indicating potential interactions.

#### 2.7.8. Transmission Electron Microscopy (TEM)

The morphology of the prepared nanoemulsion was analyzed with TEM (TFS TALOS F200X, Thermo Fisher Scientific, Waltham, MA, USA) operated at 200 kV [34]. Imaging was carried out by placing the sample directly on the carbon-coated TEM grid and incubating it for 10 s before washing for 25 times and drying it at room temperature (25 ± 2 °C).

#### 2.7.9. In Vitro Release

The release of TDL from nanoemulsion was evaluated using a vertical Franz diffusion cell (Orchid Scientific, Nashik, India), as suggested for the release testing of topical preparations [35]. The release barrier was a cellophane dialyzing membrane (MWCO 12–14 kDa), which had been soaked in deionized water overnight. The membrane was positioned between the upper and lower chambers, with an active drug release area of 0.64 cm^2^. An accurately weighed amount (1 mL) of selected nanoemulsions (contains 2 mg of TDL) was kept in the top chamber [36]. The release medium, consisting of phosphate buffer (pH 7.4) with 0.2% *w*/*v* Transcutol, was included in the receiver compartment to retain sink conditions. The system was maintained at 37 ± 0.5 °C and agitated at 100 rpm. At particular time intervals, aliquots were collected and replaced with fresh solvent. The collected samples were further filtered, diluted with the mobile phase, and analyzed for TDL by the previously described HPLC method. The release kinetics and mechanism were assessed by calculating the correlation coefficient (r^2^) using various mathematical models described in the literature [37]. All experiments were carried out in triplicate, and the resulting data are presented as the mean values with standard deviations (SDs).

### 2.8. Preparation of Tadalafil-Loaded Nanoemulgel

The optimized TDL-loaded nanoemulsion (S3) was converted into a gel formulation to enhance skin penetration and prolong drug release. The gel base was formulated by dispersing 1 g of Carbopol 934 in water to achieve a 1% *w*/*w* concentration, followed by soaking for 12 h to allow complete hydration of the polymer. A 10 g portion of the hydrated Carbopol was then neutralized with 2% triethanolamine to form a viscous gel base. The optimized nanoemulgel was prepared by incorporating 5 mL of the nanoemulsion, equivalent to 20 mg of TDL, into 5 g of the neutralized Carbopol gel. The final formulation was designed such that 1 g of the gel contained 2 mg of TDL, making it suitable for topical application.

### 2.9. Characterization of Nanoemulgel

The TDL-loaded nanoemulgel was visually assessed for color, uniformity, phase separation, pH, and viscosity. The spreadability of the prepared nanoemulgel was tested by placing 1 g of the gel on a glass plate (5 cm^2^ area), covering it with another glass plate, allowing it to spread for 5 min and the diameter was recorded [38].

### 2.10. Permeation Studies

The ex-vivo skin permeation ability of developed TDL-loaded nanoemulgel was evaluated using excised Wistar rat skin and the standard Franz diffusion technique [39]. The diffusion cell has an active permeation area of ~0.64 cm^2^, with a receiver cell volume of 5 mL, maintained at 37 ± 1 °C. Skin membranes were prepared by removing the hair, excising the dorsal skin, and removing the visceral tissue. The excised skin was stored at −20 °C until experimentation. Phosphate buffer solution (pH 7.4) and ethanol in a 50:50 ratio was used as a receptor medium to keep sink conditions. The receptor medium was stirred continuously at 150 rpm using Teflon coated magnetic bead in a magnetic stirrer. The rat skin was positioned on the Franz cells with the dermis side facing the receptor compartment, and the TDL gel (equivalent to 2 mg of drug) was placed on the donor compartment. Samples of the receptor fluid were taken at various time points, filtered, and checked for TDL content using HPLC. All experiments were conducted in triplicate, and the data are reported as the mean ± standard deviation (SD).

### 2.11. Pharmacodynamic Studies

The animal experiment procedure was approved by the Animal Ethics Committee (AEC) of Ras Al Khaimah Medical and Health Sciences University (AEC-RAKMHSU-PG-C-02-2023-2024). Adult female Wistar rats (12 weeks old; 200–250 g) were housed under controlled conditions at 22 ± 2 °C and 40–60% humidity, with a 12-h light/dark cycle, and were provided food and water ad libitum. The rats were randomly assigned into four groups (6 rats per group) as follows: Group I (n = 6): Normal control group (baseline; neither subjected to cold induction nor drug application). Group II (n = 6): Model untreated group (subjected to cold-induced vasoconstriction only). Group III (n = 6): Standard group (treated with a standard formulation: 0.2% *w*/*w* nitroglycerin topical ointment). Group IV (n = 6): TDL-treated group (received TDL-loaded nanoemulgel). The rats were placed in a fixator with their tails exposed, and TDL-loaded nanoemulgel (gel containing 2 mg of drug) was applied to the tail at room temperature (24 ± 2 °C). Following the application, the tails of the rats (excluding the normal control group) were submerged in a 10 °C water bath for 5 min to induce in-vivo vasoconstriction. Regional blood flow in the caudal arterial cortex was measured using infrared thermal imaging (Ti120, Hanmatek, Shenzhen, China) at 24 °C and again at 10 °C to confirm vasoconstriction [40,41].

### 2.12. Pharmacokinetic Studies

The adult female Wistar rats (200–250 g) utilized in the in vivo pharmacokinetic investigation were kept at room temperature (22 ± 2 °C) with a 12-h light/dark cycle. The rats were provided food and water and observed for one day. Two groups of six rats each (n = 6) were randomly selected from among all the animals. Animals were anesthetized by intraperitoneal administration of ketamine (40 mg/kg) and xylazine (5 mg/kg). In Group 1 (n = 6), the dorsal area hair was removed, and 1 g of the optimized TDL nanoemulgel (containing 2 mg of TDL) was applied uniformly to the skin. In Group 2 (n = 6), TDL suspension in 0.5% HPMC solution (1 mL, containing 2 mg of TDL) was administered orally using an intragastric gavage. The dose of 2 mg of TDL was calculated based on the human equivalent dose (10 mg), using a standard body surface area conversion equation described in the literature [42]. Blood samples (~200 µL) were withdrawn from the retro-orbital plexus at predefined time points. Each sample was transferred to dry heparinized tubes, and 1 mL of normal saline was administered via intragastric gavage after each sample collection to prevent dehydration. Plasma samples were treated with 1N HCl and 2 mL diethyl ether, then vortexed for 10 min to precipitate proteins. After centrifuging (4000 rpm) for 15 min, the top layer was removed, filtered, and dried. A 45:55 (*v*/*v*) ratio of acetonitrile: water (contains 0.1% trifluoroacetic acid) was used to dissolve the residue, and HPLC was used for analysis. Various pharmacokinetic parameters were determined using non-compartmental analysis.

### 2.13. Skin Irritation Test

The backs of two groups of six adult female Wistar rats (200–250 g) were shaved 24 h before the irritation study. Blank nanoemulgel (without TDL) was applied to the control group (n = 6), while TDL-loaded nanoemulgel was applied to the test group (n = 6) over a 9 cm^2^ hair-free area. The treated areas were visually inspected at 1 and 24 h for signs of erythema and/or edema. The skin condition was assessed using the Draize scoring criteria, which rates erythema severity on a scale of 0 to 4:0 for no erythema, 1 for slight erythema (very faint light pink), 2 for moderate erythema (dark pink), 3 for moderate to severe erythema (light red), and 4 for severe erythema (extreme redness). This method provided a standardized assessment of skin irritation [43].

### 2.14. Stability Studies

Short-time stability of the selected nanoemulsion (S3) was assessed over three months under refrigeration (2–8 °C) in an amber container. Various factors like phase separation, flocculation, precipitation, drug content (%), pH, transmittance (%), dilution potential, droplet size, PDI, zeta potential and viscosity. The TDL nanoemulgel was stored in glass vials under controlled conditions of 25 ± 0.2 °C and 75 ± 5% RH (Climate chamber, Memmert, Germany) for three months [44]. Test formulations were taken at various intervals and assessed for appearance, pH, viscosity, and drug content. All experiments were conducted in triplicate, and the results are presented as the mean ± standard deviation (SD).

### 2.15. Data Analysis

All results were reported as the mean ± SD. Student’s *t*-test or one-way ANOVA was used to assess statistical significance between groups, with a *p*-value less than 0.05 considered significant. All data were statistically analyzed with GraphPad Prism 10 (GraphPad Software, Version 10.4.1).

## 3. Results and Discussion

### 3.1. Solubility Assessment and Selection of Excipients

TDL, a BCS class II drug, with solubility values that range from 0.73 μg/mL [45] to 3.48 μg/mL [46], making it nearly insoluble in water. Therefore, the selection of oil, surfactant, and cosurfactant is crucial for the fabrication of a nanoemulsion. This research objective was to develop an o/w nanoemulsion for the transdermal delivery of TDL, designed to permeate through the stratum corneum. Thus, it was essential to identify pharmaceutically acceptable solvents, surfactants, cosurfactants, and oils, and optimize their ratios to ensure product stability. The solubility of the drug (TDL) in various oils is presented in Table 1. The maximum solubility of drug was detected in cinnamon oil (53.55 ± 2.58 mg/mL), while isopropyl myristate exhibited the lowest solubility (0.11 ± 0.02 mg/mL) among the tested compounds. Cinnamon oil was selected for its high TDL solubility and therapeutic properties, such as antioxidative, anti-inflammatory effects, and inhibition of vascular smooth muscle growth, platelet activity, thrombosis, and angiogenesis, which may synergize with TDL’s action in a nanoemulsion [47]. Higher drug solubility in the oil phase offers several formulation advantages, including enhanced drug loading capacity, improved stability, reduced oil and surfactant requirements, and improved bioavailability [48]. Additionally, efficient solubilization promotes better drug dispersion and absorption, contributing to improved bioavailability, especially for poorly water-soluble drugs. This also enables more controlled and predictable drug release profiles.

Similarly, Cremophor RH40 (surfactant; solubility ~27.56 ± 1.23 mg/mL) and Transcutol (cosurfactant; solubility ~33.38 ± 1.32 mg/mL) were chosen for nanoemulsion preparation due to their high TDL solubility. The effectiveness of these selected vehicles in nanoemulsion formulation is well-documented in the literature. For instance, self-nano-emulsifying drug delivery systems made considerable use of hydrophilic non-ionic surfactants like Cremophor RH40 which had a relatively high HLB [49]. Transcutol is authorized for topical and transdermal use at up to 50% concentrations worldwide. Additionally, Transcutol is a protic solvent/cosolvent that is compatible with both polar and non-polar compounds and is biocompatible, biodegradable, optically clear, transparent, and odorless. The skin penetration characteristics of Transcutol^®^ in complex preparations, such as nanoemulsions, are well-established [50,51]. Selecting a surfactant with the appropriate HLB value is essential for nanoemulsion stability. For o/w nanoemulsions, an HLB value above 10 is required; therefore, a balanced combination of low and high HLB surfactants reduces interfacial energy and enhances overall thermodynamic stability [52]. It was reported that when formulating nanoemulsions using natural oils, an HLB value more than 10 typically leads to o/w nanoemulsions, while a value less than 10 generally forms w/o nanoemulsions [53].

**Figure 2:**
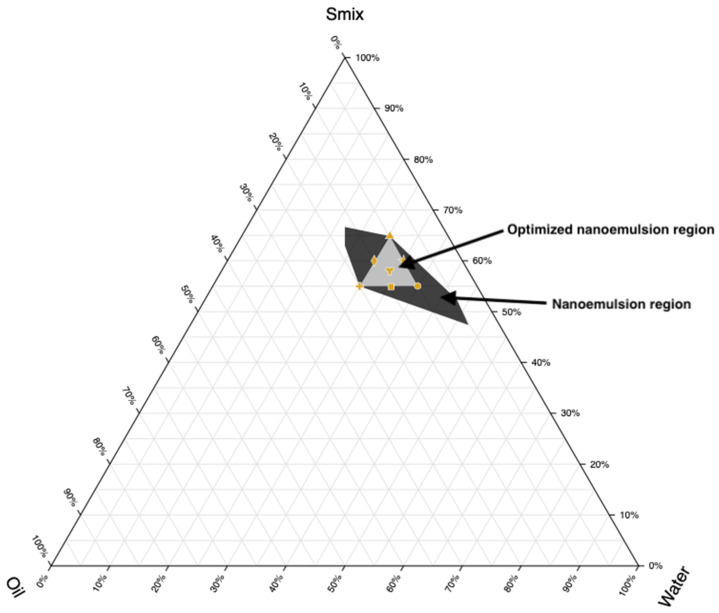
Ternary phase diagram depicting the nanoemulsion region and optimized nanoemulsion region prepared using cinnamon oil, Transcutol/Cremophor RH 40 (S_mix_), and water.

Based on the above considerations, Cremophor RH 40 (HLB 15) and Transcutol (HLB 4.2) were mixed at a 1:0.7 Smix ratio to achieve an optimal required HLB value of 10.55, which is ideal for o/w nanoemulsion formation. It has been demonstrated that the selection and use of an appropriate blend of a low HLB surfactant (Carbitol) and a high HLB surfactant (Tween 80) facilitates the formation and stabilization of Ropinirole o/w nanoemulsions upon dilution with water [52]. The binodal curve, which separates the two-phase region from the single-phase region in the diagram, was determined by visually observing changes in the sample’s appearance, specifically the transition between cloudy and clear states. To complete the entire nanoemulsion domain, fixed Smix ratio (1:0.7) was used with varying ratios between oil and water ranging from 1:9, 2:8, 3:7, 4:6, and 5:5. The phase diagram for the systems comprising the surfactant: cosurfactant (Cremophor RH40: Transcutol) blend (Smix), along with the oil phase as well as water, is depicted in Figure 2. A triangular area was randomly chosen and designated as a triangle within the nanoemulsion region (Figure 2), wherein formulations S1–S7 were developed.

The composition of components in formulations must sum to 100%. Based on Figure 2, points were selected at key positions: the three vertices, three midpoints between vertices, and the center point. Every vertex denotes a formulation where one component is at its highest concentration and the others are at its lowest. Midpoints between vertices show formulations with an average concentration of the two relevant components, while the center point denotes a formulation with equal amounts of each component. Seven nanoemulsion formulations (S1–S7) were selected for further study, and their compositions are detailed in Table 2.

Ultrasonication was performed for 5 min at a frequency of 20 kHz based on preliminary optimization studies, which evaluated various sonication times (2–10 min) and frequencies. These preliminary trials demonstrated that a 5 min duration at 20 kHz consistently produced nanoemulsions with the smallest droplet size and an acceptable PDI, indicating uniformity in droplet distribution. Longer sonication times did not yield significant improvements in droplet size and posed a risk of thermal degradation, while shorter durations resulted in incomplete emulsification. The significance of ultrasonic frequency and duration in the preparation of nanoemulsions has been well documented in the literature [54,55]. The use of a cyclomixer prior to sonication ensured initial homogenization, thereby enhancing the efficiency of droplet size reduction during ultrasonication.

### 3.2. Characterization of Nanoemulsion

Characterization of nanoemulsions is crucial to ensuring their stability, efficacy, and suitability for pharmaceutical applications. The physicochemical properties of nanoemulsions (S1–S7) were assessed and summarized in Table 3. Drug content (%) ensures uniform distribution, while pH affects stability and compatibility. Drug content ranged from 90% to 105%, and the formulations had a pH of approximately 5–6, aligning with skin pH, suggesting a minimal risk of skin irritation. Transmittance (%) indicates clarity and dilution potential assesses stability upon dilution. Droplet size (nm) and PDI influence drug release, bioavailability, and physical stability, while zeta potential (mV) predicts electrostatic stability, preventing aggregation, and viscosity (cP) affects flow properties and administration [28]. A high percentage of transmittance (>90%) confirmed transparency and nanometer-sized droplets (92.9–197.5 nm), with no phase separation observed upon 10-fold dilution. The polydispersity index (PDI) values (0.347–0.856) indicated uniform, homogeneous spherical droplets. Zeta potential values (0.00 mV) suggested low repulsion, with no significant effect from formulation components, potentially stabilizing the dispersion likely due to the presence of steric hindrance provided by surface-active agents adsorbed onto the droplet surface. Non-ionic surfactants, such as Cremophor RH40, contribute to the generation of a stable hydrophilic shell around the droplets, forming a steric barrier that prevents coalescence and aggregation despite the near-neutral surface charge [56]. This indicates that steric stabilization, rather than electrostatic repulsion, plays a dominant role in preventing droplet aggregation, even in the absence of significant surface charge, ensuring the physical stability of the formulation.

Viscosity (62.5–65 cP) ensured ease of topical application and retention. Results indicated the unique structural and physicochemical properties of the prepared nanoemulsion. It was reported that a kinetically stable nanoemulsion with droplets smaller than 500 nm has become a sophisticated drug delivery system, enhancing solubility, bioavailability, and targeted delivery of therapeutics [57].

### 3.3. Thermodynamic Stability Studies

Thermodynamic stability is crucial for preserving the integrity of the nanoemulsion system. Stability assessments of all nanoemulsion batches (S1–S6) showed no signs of instability, except for S7, which exhibited instability during the freeze-thaw cycle test (Table 4). Batch S7 demonstrated nano-level aggregation post-freeze-thaw, even though macroscopic phase separation was not observed during the heating-cooling cycle and centrifugation tests. This instability may be attributed to excipient crystallization at low temperatures or phase transitions during the freeze-thaw cycle, leading to a weakened droplet interface and increased susceptibility to destabilization. This observation is consistent with previous findings indicating that the sequence of crystallization events and the physical state of surfactants at the oil–water interface play a critical role in determining the freeze–thaw stability of emulsions [58].

During the heating–cooling cycle, no phase separation, creaming, or changes in appearance were observed. The centrifugation test confirmed physical stability under stress, with no phase separation. Similarly, the freeze-thaw cycle revealed no aggregation, phase separation, or alterations in texture or consistency, indicating robust stability across all tests. Additionally, particle size as well as PDI were determined before and after the freeze-thaw test to identify the most stable batch among the selected nanoemulsions, as shown in Table 5. Among all the evaluated nanoemulsions, S1, S3, and S6 exhibited the smallest globule size with acceptable PDI in both pre-thaw and post-thaw tests. In addition, the variation of globule size was lowest with S3. The minimal variation in globule size observed for formulation S3 between pre-thaw and post-thaw conditions (91.30 ± 0.64 nm to 99.10 ± 0.78 nm) can be attributed to its optimized composition of Smix (55.04%), oil (10.1%), and water (34.86%), which appears to provide enhanced thermodynamic stability compared to S1 and S6. The relatively higher water content in S3, as compared to S1 (25.22%) and S6 (30%), likely facilitates better dispersion of the oil phase and more efficient solubilization of the surfactant mixture. This helps in preventing globule aggregation or coalescence during freeze-thaw stress. It was reported that nanoemulsions with an optimal balance of water and surfactant/oil ratio tend to form a more robust interfacial film around droplets, increasing their resistance to thermal and mechanical stress. Furthermore, the Smix content in S3 (55.04%) is sufficiently high to stabilize the oil droplets, yet not excessive like in S1 (64.87%), where higher surfactant concentration may lead to phase inversion or destabilization upon freezing. As for S6, although the Smix and oil concentrations are similar to S3, the lower water content may result in a more viscous system with less flexibility in droplet rearrangement, thus leading to a pronounced increase in droplet size after thawing (from 114.40 ± 1.34 nm to 197.80 ± 0.42 nm). In summary, the enhanced stability of S3 during freeze-thaw cycling is likely due to its optimized balance of oil, Smix, and water, promoting a stable interfacial layer and minimal phase disruption. This observation aligns with previously published findings on nanoemulsion stability under thermal stress [59,60].

**Figure 3:**
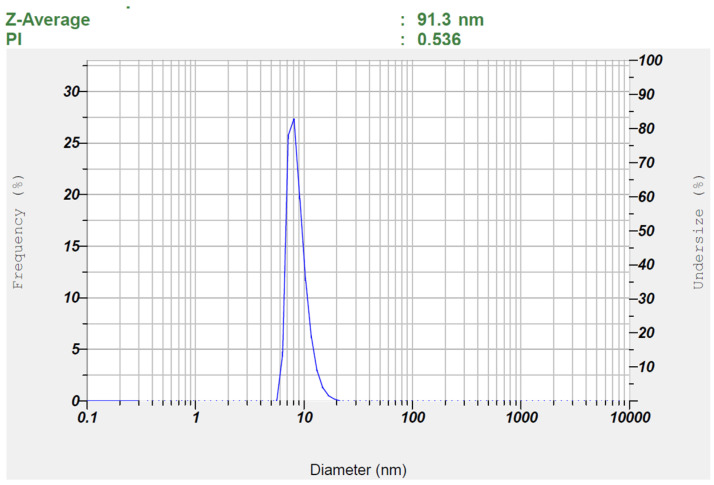
Measured particle size of the optimized TDL-loaded nanoemulsion (S3).

**Figure 4:**
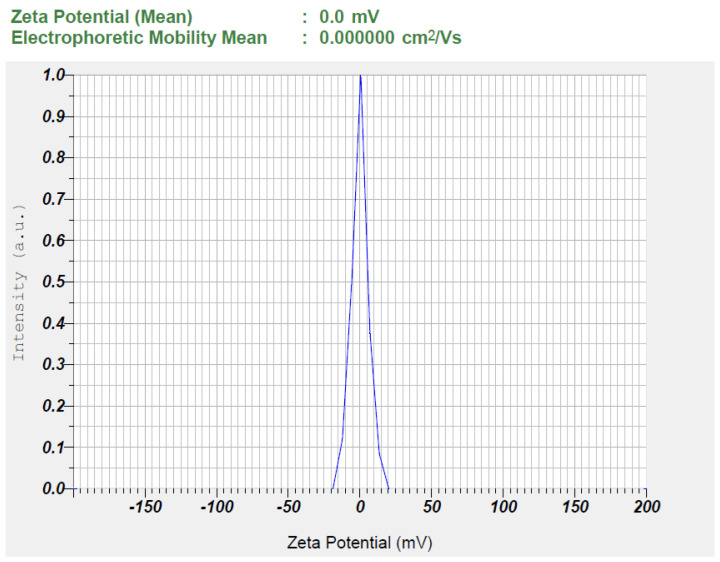
The observed zeta potential of the optimized TDL-loaded nanoemulsion (S3).

Figure 3 and Figure 4 show representative images of batch S3’s particle size and zeta potential, respectively. It is obvious from Figure 3 that the distribution of globule size is narrow as well as unimodal. Nanoemulsions with a uniform and small droplet size significantly enhance transdermal drug delivery by improving skin penetration and hence bioavailability. Their small particle size and enormous surface area ensure close association with the skin, resulting in improved interaction with skin lipids and facilitating efficient diffusion through the stratum corneum [61]. Additionally, the narrow particle size distribution minimizes the risk of Ostwald ripening and coalescence, thereby enhancing the long-term stability of nanoemulsions [62].

### 3.4. FTIR Studies

**Figure 5:**
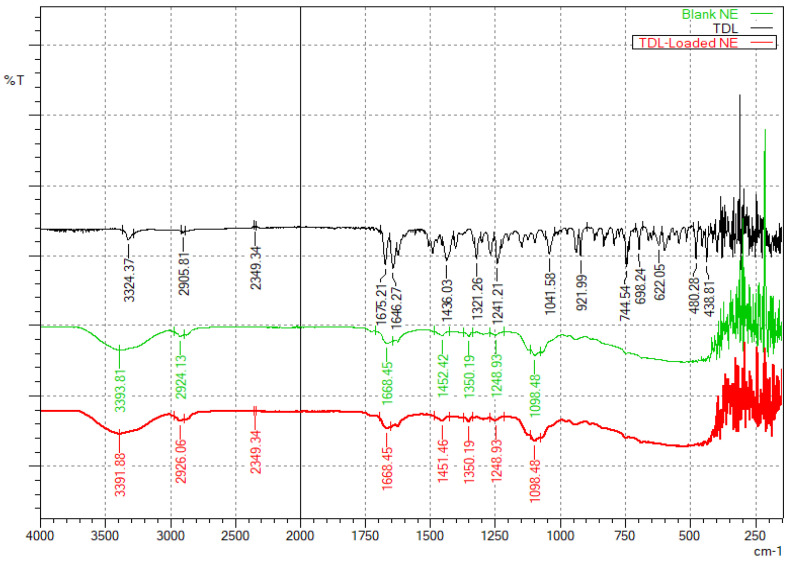
FTIR spectra of TDL, a blank nanoemulsion, and the TDL-loaded nanoemulsion (S3).

The FTIR spectra provide insights into the molecular interactions between TDL, and the excipients used to formulate nanoemulsions. The spectrum (Figure 5) of pure TDL exhibits characteristic spectral peaks at 3324.37 cm^−1^ (N-H stretching, amide), 2905.81 cm^−1^ (C-H, aliphatic stretching), 1646.27 cm^−1^ (C=O, amide stretching), 1321.26 cm^−1^ and 1241.21 cm^−1^ (C-N stretching), and various aromatic bending vibrations. These spectral features are consistent with a previous report, wherein characteristic TDL peaks were observed at 3324.7 cm^−1^ (N–H stretching of the secondary amine group), 2904.3 cm^−1^ (C–H stretching of aliphatic CH_3_), 1674.7 cm^−1^ (C=O stretching), and 1644.7 cm^−1^ corresponding to C=C stretching of the aromatic ring [23]. Similarly, another study has noted corresponding peaks at 3327 cm^−1^, 2906 cm^−1^, 1678 cm^−1^, and 1649 cm^−1^ for N–H, C–H, C=O, and C=C stretching vibrations, respectively [63]. The close agreement between the observed peaks and literature values confirms the structural integrity of TDL in the formulation. The blank nanoemulsion containing cinnamon oil, Transcutol, and Cremophor RH40 shows a broad O-H, stretching peak at 3393.81 cm^−1^, C-H, aliphatic stretching at 2924.13 cm^−1^, and C=O stretching at 1668.45 cm^−1^, likely from the excipients. Additionally, the presence of C-O and aromatic C=C stretching suggests contributions from the surfactants and cinnamon oil. The drug-loaded nanoemulsion retains most of TDL’s functional groups but with slight shifts in peaks, particularly at 3391.88 cm^−1^ (broadened O-H/N-H, due to stretching) and 1668.45 cm^−1^ (C=O, stretching). These shifts indicate potential hydrogen bonding and encapsulation effects, confirming the successful entrapment of TDL within the nanoemulsion system. Importantly, the absence of new peaks suggests that TDL remains chemically stable without undergoing significant interactions with the excipients.

### 3.5. TEM

**Figure 6:**
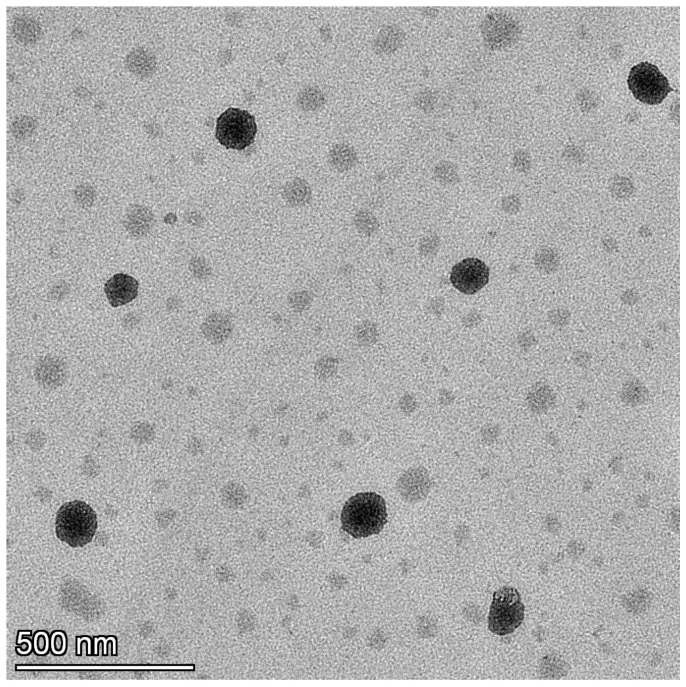
TEM image of the TDL-loaded nanoemulsion (S3).

The TEM image of the TDL nanoemulsion (S3) is presented in Figure 6. The figure clearly shows that the system contains uniformly sized, circular droplets that are easily distinguishable. The scale bar in the image represents 500 nm, and individual particles were measured relative to this scale. The TEM analysis revealed that the particle sizes were visually estimated to be in the range of approximately 90–100 nm. This is in good agreement with the particle size obtained from dynamic light scattering (DLS), which showed 91.30 ± 0.64 nm before thawing and 99.10 ± 0.78 nm after thawing. The slight variation between the two techniques is attributed to the measurement principles, where DLS reports the hydrodynamic diameter while TEM reflects the dry particle size. The droplets are visible as dark structures against a bright background, randomly distributed throughout the field with no indication of agglomeration. This observation is consistent with findings from similar studies, where TEM images of nanoemulsions displayed spherical, uniformly dispersed droplets appearing dark against a bright background [64].

### 3.6. In Vitro Drug Release

**Figure 7:**
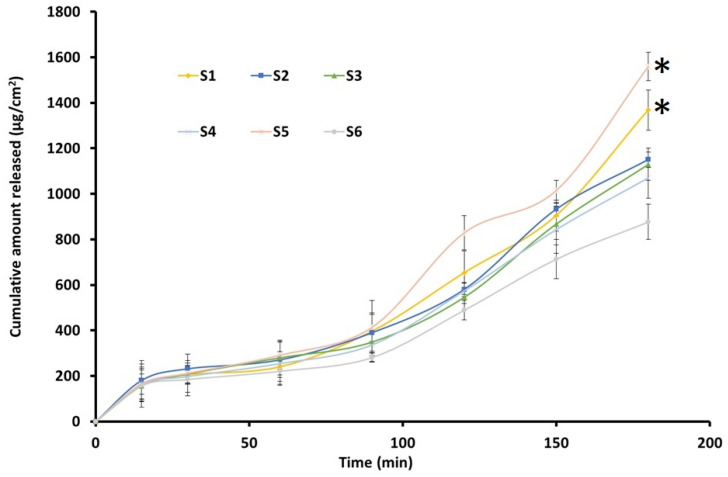
Release of drug from the prepared TDL-loaded nanoemulsion (S1–S6) (n = 3). * Statistically significant from other formulations at *p* < 0.05.

The drug release from nanoemulsions is crucial for absorption and therapeutic efficacy. Figure 7 illustrates the cumulative release of TDL plotted over various time intervals from formulations S1–S6. All nanoemulsion formulations exhibited a steady release, exceeding 90% within 3 h. The release profiles were comparable in the first 90 min, while the cumulative amount varied at 3 h. The highest drug release was observed for S5 and decreased as S1 > S2–S3 > S4 > S6. However, no significant difference in drug release was observed between the different nanoemulsion formulations. The sustained release profiles suggest that nanoemulsions can prolong drug availability, thereby enhancing the therapeutic potential of TDL. Release kinetics analysis indicated that the drug followed a zero-order model with a high r^2^ value (0.9142). The zero-order drug release from nanoemulsion droplets indicates a sustained and controlled release, where the drug is delivered at a constant rate, ensuring a steady therapeutic effect over time [65]. This suggests that nanoemulsion droplets function as controlled reservoirs, releasing the drug independently of concentration gradients in the surrounding medium. This enhances drug stability and bioavailability, maintaining consistent drug levels and minimizing fluctuations that could lead to toxicity or subtherapeutic effects. This release profile is often desirable for controlled drug delivery, ensuring gradual and sustained therapeutic levels [66]. Formulation S3 was selected for nanoemulgel preparation based on low particle size, median in vitro release profile, and consistent vesicle size in repeated freeze-thaw tests.

### 3.7. Characterization of Nanoemulgel

To enhance skin retention, TDL-loaded nanoemulsions were incorporated into an aqueous gel formulation using carbopol polymer. The resulting nanoemulgel exhibited a smooth, uniform, and transparent appearance, with no phase separation, confirming its stability. The pH of the formulation (5.68 ± 0.08) was close to skin pH, ensuring minimal skin irritation and improved patient compliance [67]. It also demonstrated high drug content (98.64 ± 0.77%), indicating efficient drug loading. Viscosity (1030.67 ± 12 cPs) and spreadability (4.62 ± 0.25 cm) were well-balanced, ensuring ease of application, good consistency, and improved patient compliance, as previously reported [68]. These findings confirm the suitability of the nanoemulgel for topical administration, aligning with previously reported formulations.

### 3.8. Ex Vivo Permeation Studies

**Figure 8:**
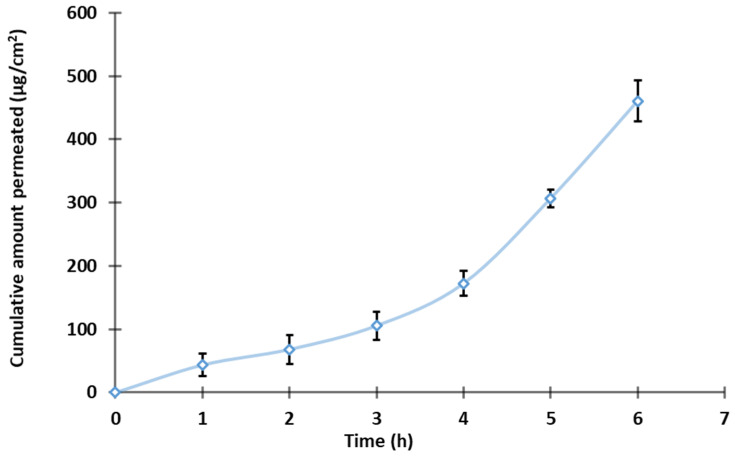
Permeation profile of the TDL-loaded nanoemulgel (n = 3).

The diffusion of bioactives across biological membrane barriers is primarily influenced by the drug’s physicochemical properties, the membrane’s physiological characteristics, and available transport routes. Figure 8 presents TDL permeation across an isolated rat skin from S3-loaded nanoemulgel. Optimized formulation exhibited a typical permeation profile with high steady-state flux values (71.85 μg/cm^2^/h), indicating that S3 possesses suitable physicochemical properties for transdermal permeation. In addition, a short lag time (0.7 h) and good permeability coefficient (Kp = 3.59 × 10^−2^ cm/h) were exhibited by the developed nanoemulsion gel. The above observation indicates the potential of the developed formulation to quickly transport through the skin membrane. The enhanced permeation characteristics can be attributed to several factors. The nano-size of the vesicles provides greater surface area which in turn helps in easy drug absorption, facilitating a closer connection with the stratum corneum and promoting efficient drug diffusion through the skin layers [69]. Incorporating surfactants such as Cremophor and Transcutol into the nanoemulsion formulation disturbs the lipid bilayers of the stratum corneum, augmenting skin permeability by reducing the barrier resistance and allowing for improved drug penetration [50,70]. Moreover, the inclusion of these agents in the nanoemulsion can fluidize the lipid structure of the stratum corneum, thus facilitating drug permeation. Collectively, these factors contribute to the superior transdermal delivery performance observed with the S3 nanoemulsion gel formulation.

### 3.9. In-Vivo Pharmacodynamic Studies

Rats are used in pharmacology experiments to study cold-induced vasoconstriction due to their physiological similarity to humans, controlled experimental conditions, and ease of handling. Their microcirculatory response to cold exposure is well-defined, making them ideal for evaluating vasodilators and new drug formulations [71]. Additionally, their thin skin and small size allow for precise measurement of blood flow changes using thermal imaging or other vascular assessment techniques, supporting research on treatments for conditions like RP [72]. Several studies have consistently reported a higher incidence of RP among females [1,4,5]. This sex-based predisposition has been taken into consideration while designing our experimental model. Accordingly, female Wistar rats were selected for the study to better replicate the clinical features and pathophysiological relevance of RP observed predominantly in women. Infrared thermal imaging is a non-invasive, non-contact visualization technique that relies on infrared radiation [73]. It operates in real-time, capturing images based on the radiation or energy emitted, provided the system’s temperature is above absolute zero. Infrared thermal imaging has been utilized in both animal studies [41] and human applications [74,75]. The primary determinant of an object’s infrared radiation intensity is its temperature; thus, when temperature increases there will be higher infrared energy release as well. For instance, the infrared method is used to detect near-surface body tumors, such as breast cancer, as these tumors typically exhibit increased blood supply, leading to elevated skin temperature in the affected area [76]. In the present technique, infrared thermography analyzes surface skin temperature variations caused by vasodilation from TDL or standard nitroglycerin treatment and cold-induced vasoconstriction, creating a thermal contrast that is captured by an infrared camera.

**Figure 9:**
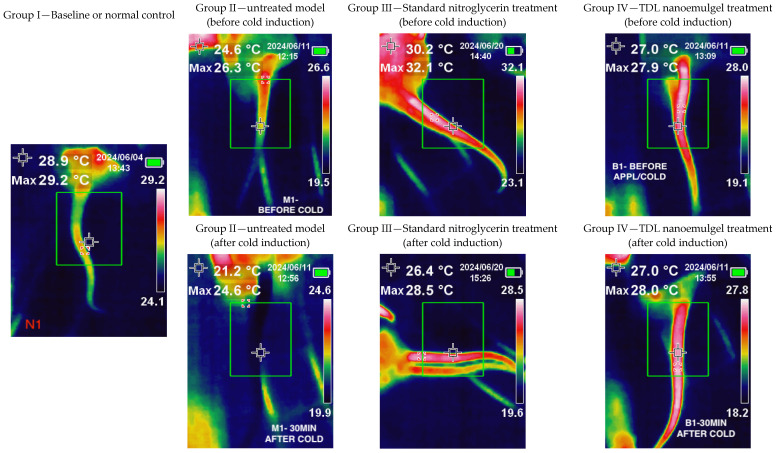
Results of the pharmacodynamic study of the TDL-loaded nanoemulgel.

The color changes between affected and unaffected skin in RP are clearly distinguishable, as outlined in the International Consensus Criteria for the diagnosis of RP [77]. The thermal scanning of Group I (Normal control group) showed a greenish image since the animals were not subjected to either cold induction or drug application (Figure 9). The temperature displayed in the upper left corner (28.9 °C) represents the sensor reading at the marked area of the tail. The maximum and minimum temperatures recorded in the tail region were 29.2 °C and 24.1 °C, respectively, indicating a relatively uniform temperature distribution along the tail that closely approximated ambient room temperature. Group II animals (Model untreated group) exhibited a bluish-colored tail due to cold-induced vasoconstriction. The temperature displayed in the upper left corner (24.6 °C) corresponds to the sensor reading at the marked tail region prior to cold induction, while the post-induction temperature reading was 21.2 °C. This reduction in temperature clearly reflects the vasoconstrictive response triggered by exposure to ice-cold water, resulting in decreased blood flow to the area. Thermal images of Group III (standard group treated with nitroglycerin 0.2% *w*/*w* topical ointment) and Group IV (TDL nanoemulgel-treated group) showed red coloration, indicating increased blood flow after vasoconstriction was induced by ice-cold water and the prior application of the formulation. The thermal imaging observations indicate that both nitroglycerin ointment and the TDL nanoemulgel effectively counteract cold-induced vasoconstriction, maintaining increased blood flow even 30 min post-cold exposure. In the standard treatment group, the temperature recorded at the marked tail region prior to cold induction was 30.2 °C; however, it returned to 26.4 °C, a value closer to the original baseline temperature. In contrast, animals treated with the TDL nanoemulgel maintained a consistent temperature of 27 °C both before and after cold induction, indicating the formulation’s superior efficacy in counteracting cold-induced vasoconstriction and preserving peripheral blood flow. This suggests that the TDL nanoemulgel may serve as a potent vasodilator, comparable to standard nitroglycerin ointment, in mitigating reduced blood flow resulting from cold-induced vasoconstriction. TDL nanoemulgel may offer advantages over standard nitroglycerin ointment due to its enhanced skin penetration, prolonged therapeutic action, and improved bioavailability. The nanoemulsion-based formulation facilitates deeper and more sustained drug delivery, potentially reducing the frequency of application required for effective vasodilation [78]. Additionally, TDL nanoemulgel may minimize the side effects commonly associated with nitroglycerin, like headaches, allergic reactions, palpitations, and hypotension, making it a more patient-friendly alternative for managing cold-induced vasoconstriction. However, it is important to note that while nitroglycerin ointment has shown promise, its exact role in the management of Raynaud’s phenomenon is not known, and further robust investigations are warranted to fully understand its efficacy and safety profile [79].

### 3.10. In Vivo Pharmacokinetic Studies

**Figure 10:**
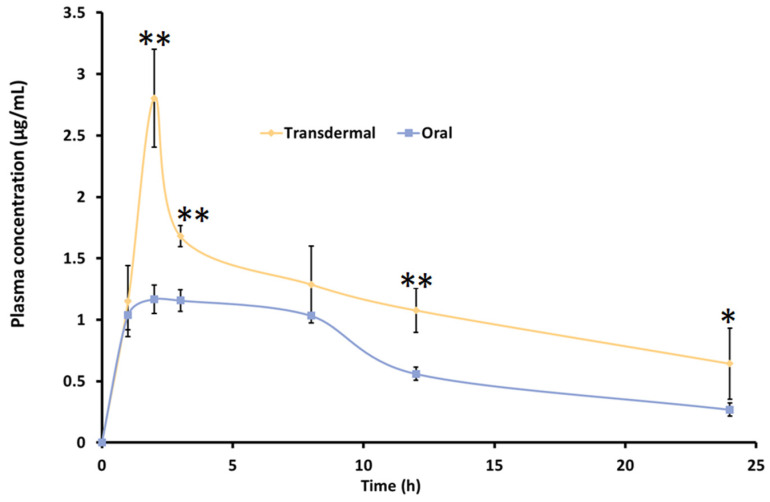
Pharmacokinetic profile of TDL-loaded nanoemulgel (transdermal) and TDL suspension (oral) in rats. Values are presented as the mean ± SD of six animals. Statistical significance versus oral, ** indicates *p* < 0.0001, and * indicates *p* < 0.05.

Animal studies are crucial for understanding drug pharmacokinetics, optimizing dosage regimens, ensuring safety, and supporting product development, especially for clinical use. To assess the potential of the formulated nanoemulgel for transdermal therapy, an in vivo study was conducted in Wistar rats. Pharmacokinetic parameters were compared between transdermal and oral administration using a 2 mg/rat dose of TDL. The resulting plasma drug concentration profiles for both routes are illustrated in Table 6. The pharmacokinetic profile of TDL marginally varied between transdermal and oral administration (Figure 10). The plasma drug levels noticed here are consistent with findings from the previous study [13]. The quick appearance of TDL in plasma in 1 h may be due to the excellent permeation potential of TDL into and across the skin, facilitating rapid absorption, as noted in previous studies [80,81]. This would be particularly beneficial for Raynaud’s phenomenon, wherein the rapid dilation of blood vessels following vasospastic episodes could help restore oxygenated blood flow to ischemic tissues, thereby reducing the risk of tissue damage and associated complications.

The plasma concentration gradually increased over time, with transdermal administration showing T_max_ (2 h) comparable to oral delivery. Notably, the C_max_ and AUC were significantly different (*p* < 0.05) with the nanoemulgel compared to the oral suspension, as shown in Table 6. The C_max_ value observed in the present study (2.13 ± 0.21 µg/mL) was approximately tenfold higher compared to the values reported for oral jellies, 165.2 ± 7.9 ng/mL, and self-nanoemulsifying chewable tablets, 125.2 ± 6.6 ng/mL [24,82]. This substantial increase suggests enhanced absorption, likely due to more efficient drug transport across the skin membrane. Similarly, the T_1/2_ obtained for TDL-loaded nanogel (16.12 h) was more statistically significant than its counterpart, the TDL oral suspension, value of 4.53 h (*p* < 0.0001). It is worthwhile to note that clinical evaluation in a patient with secondary RP confirmed that TDL, at an equipotent dose compared to other PDE-5 inhibitors such as sildenafil, significantly improved capillary blood flow and resulted in fewer Raynaud’s attacks. This effect is likely due to the longer half-life of TDL compared to the shorter half-life of sildenafil (3.8 h), which may contribute to more sustained therapeutic benefits [83]. The bioavailability (AUC_0–24_) of TDL was moderately higher with transdermal therapy than the oral administration. The improvement in drug availability with transdermal therapy could be attributed to factors such as bypassing the first-pass effect and the superior permeation ability of nanoemulsion [84]. Nanoemulgel also demonstrates sustained-release behavior compared to oral nanosuspension, improving treatment efficacy for Raynaud’s syndrome by ensuring prolonged drug availability at the target location, reducing dosing frequency, and minimizing systemic side effects

### 3.11. Dermal Irritation Test

The dorsal skin of the rats was shaved 24 h before the irritation test to prevent any bias from shaving-related injuries. After this period, TDL nanoemulgel and blank nanoemulgel were applied accordingly, and the animals were evaluated based on Draize scoring criteria [43] at 1 h and 24 h. There was no indication of erythema or edema in either group (Table 7). The absence of these reactions suggests that the nanoemulgel is well-tolerated and suitable for transdermal application.

### 3.12. Stability Studies

Both the nanoemulsion (S3) and nanoemulgel (S3) remained stable throughout the three-month study, indicating their potential for extended shelf life and clinical use. The appearance of the S3 formulation did not change, as a clear solution was maintained for three months. The absence of instability indicators such as phase separation, pH fluctuations, viscosity changes, and drug content variations confirm the formulations’ suitability for pharmaceutical development. Similarly, the gel formulation was found stable and has no variation in the parameters tested. These results support their feasibility for transdermal drug delivery, ensuring consistent therapeutic efficacy and safety over time.

## 4. Conclusions

The aqueous titration method, guided by a ternary phase diagram, was utilized to develop a TDL nanoemulsion incorporating cinnamon oil as the oil phase and an optimized surfactant–cosurfactant (Smix) ratio (1:0.7) consisting of Cremophor RH40 (surfactant) and Transcutol (cosurfactant). The optimized nanoemulsion (S3) contained 10.1% oil, 55.04% Smix, and 34.86% water, exhibiting a spherical morphology, nano-sized droplets (~92 nm), a neutral zeta potential (0.0 mV), and enhanced steady-state flux (71.85 μg/cm^2^/h). FTIR analysis confirmed the absence of drug–excipient interactions, while TEM imaging revealed uniformly dispersed vesicles with no agglomeration. To improve skin application, the nanoemulsion was incorporated into a carpool-based gel, which was found to be smooth, uniform, and transparent, making it suitable for transdermal therapy. High ex vivo permeation observed here indicates its potential as an effective TDL carrier system. Pharmacodynamic studies demonstrated that transdermal application of the TDL nanoemulgel effectively mitigated cold-induced vasoconstriction, maintaining blood flow comparable to standard nitroglycerin topical ointment. Pharmacokinetic evaluation revealed significantly higher (*p* < 0.001) drug absorption by transdermal therapy than by oral nanosuspension delivery. The developed TDL nanoemulgel demonstrates strong potential for transdermal therapy, offering once-daily application and comparable pharmacokinetic and pharmacodynamic effects. These findings highlight its viability as a promising alternative to oral therapy for RP. However, the study has certain limitations, including the use of thermal imaging instead of the more precise laser doppler flowmetry for assessing blood flow and its reliance on a cold-induced rat model, which does not fully capture the complexity of the human condition. Thus, further validation in clinical settings is warranted.

Future studies should involve human subjects, utilize laser doppler imaging, and explore the formulation’s application in other vasoconstrictive conditions, such as acrocyanosis. Evaluating larger sample sizes and non-cold-induced models may further establish its benefits, including faster vasodilation, symptom relief, improved quality of life, and a reduced risk of complications such as tissue necrosis, digital ulcers, gangrene, and functional impairment.

## Figures and Tables

**Table 1:** Solubility of Tadalafil in various solvents.

Component	Solubility (mg/mL)
Oil	
Miglyol^®^ 812N	0.33 ± 0.01
Miglyol^®^ 840	0.29 ± 0.01
Miglyol^®^ 829	0.91 ± 0.14
Triacetin	3.83 ± 0.07
Isopropyl myristate	0.11 ± 0.01
Castor oil	0.45 ± 0.03
Oleic acid	0.19 ± 0.01
Cinnamon oil	53.55 ± 2.58
N-methyl pyrrolidone	325.00 ± 4.56
Surfactant/Cosurfactant	
Tween 80	0.28 ± 0.01
Span 80	0.17 ± 0.03
Cremophor RH 40	27.56 ± 1.23
PEG-400	13.97 ± 1.01
Propylene glycol	0.84 ± 0.05
Isopropyl alcohol	0.51 ± 0.01
Transcutol	33.38 ± 1.32

**Table 2:** Formulation composition used for preparing nanoemulsions with an Smix ratio of 1:0.7.

Formulations	Formulation Components		
	Smix (%)	Oil (%)	Water (%)
S1	64.87	9.91	25.22
S2	55	20	25
S3	55.04	10.1	34.86
S4	60	15	25
S5	54.91	14.7	30.39
S6	60	10	30
S7	58.1	13.33	28.57

**Table 3:** Physicochemical characteristics of the prepared nanoemulsions.

Parameter	S1	S2	S3	S4	S5	S6	S7
Drug content (%)	96.62 ± 2.05	95.16 ± 1.25	98.64 ± 0.77	95.61 ± 1.32	96.92 ± 1.64	97.58 ± 0.95	96.88 ± 1.01
pH	6.13 ± 0.11	5.68 ± 0.05	5.67 ± 0.07	6.00 ± 0.10	5.63 ± 0.08	6.09 ± 0.10	5.63 ± 0.07
Transmittance (%)	95.8 ± 1.31	98.70 ± 0.60	98.40 ± 0.26	97.00 ± 0.50	98.60 ± 0.45	97.80 ± 0.74	96.10 ± 1.80
Dilution potential	>10 fold	>10 fold	>10 fold	>10 fold	>10 fold	>10 fold	>10 fold
Droplet size (nm)	92.90 ± 2.26	195.70 ± 0.78	91.30 ± 0.64	105.50 ± 3.82	133.00 ± 0.42	114.40 ± 1.34	131.30 ± 0.90
Polydispersity index	0.67 ± 0.03	0.40 ± 0.05	0.54 ± 0.03	0.65 ± 0.00	0.43 ± 0.00	0.64 ± 0.05	0.49 ± 0.02
Zeta potential (mV)	0.0	0.0	0.0	0.0	0.0	0.0	0.0
Viscosity (cP)	64.20 ± 0.47	63.60 ± 0.20	63.10 ± 0.70	62.50 ± 0.10	65.00 ± 0.15	63.87 ±0.15	62.70 ± 0.25

Data are presented as the mean ± SD (n = 3).

**Table 4:** Thermodynamic stability studies of the prepared nanoemulsions.

Batches	Thermodynamic Stability Studies		
	Centrifugation	Heat-Cool	Freeze-Thaw
S1	√	√	√
S2	√	√	√
S3	√	√	√
S4	√	√	√
S5	√	√	√
S6	√	√	√
S7	√	√	X

Data are presented as the mean ± SD (n = 3); √: Stable (no phase separation, creaming, or changes in appearance) X: Unstable (phase separation, creaming, or changes in appearance).

**Table 5:** Globule size and PDI of selected nanoemulsions before and after thawing.

Parameter	Pre-Thaw		Post-Thaw	
Batch	Vesicle Size (nm)	PDI	Vesicle Size (nm)	PDI
S1	92.90 ± 2.26	0.856 ± 0.13	139.20 ± 0.57	0.596 ± 0.00
S2	195.70 ± 0.78	0.334 ± 0.01	197.50 ± 0.42	0.347 ± 0.00
S3	91.30 ± 0.64	0.536 ± 0.02	99.10 ± 0.78	0.550 ± 0.07
S4	105.50 ± 3.82	0.801 ± 0.33	139.90 ± 2.26	0.511 ± 0.01
S5	133.00 ± 0.42	0.490 ± 0.02	140.90 ± 0.57	0.386 ± 0.02
S6	114.40 ± 1.34	0.709 ± 0.03	197.80 ± 0.42	0.543 ± 0.02

Data are presented as the average ± SD (n = 3).

**Table 6:** In vivo parameters of TDL-loaded nanoemulgel (transdermal) and TDL suspension (oral) in rats (n = 6). * Statistically significant at *p* < 0.05.

Parameters	TDL Nanoemulgel (Mean ± SD)	TDL Suspension (Mean ± SD)
C_max_ (µg/mL)	2.13 ± 0.21	1.17 ± 0.12 *
T_1/2_ (h)	16.12	4.53
T_max_ (h)	2	2
AUC_0–24_ (µg·h/mL)	26.60 ± 3.81	16.41 ± 2.13 *

**Table 7:** Dermal irritation scores for the TDL nanoemulgel and control formulation in rats (n = 6).

	Erythema Score	Edema Score	Total Score	Irritation Potential
TDL nanoemulgel	0	0	0	None
Blank nanoemulgel	0	0	0	None

Erythema score: Degree of skin redness measured on a scale from 0 (no erythema) to 4 (severe erythema). Edema score: Degree of swelling measured on a scale from 0 (no edema) to 4 (severe edema). Total score: Sum of erythema and edema scores.

## Data Availability

The data presented in this study are contained within this article.
